# Medications for sleep disturbance in children and adolescents with depression: a survey of Canadian child and adolescent psychiatrists

**DOI:** 10.1186/s13034-020-00316-8

**Published:** 2020-03-10

**Authors:** Addo Boafo, Stephanie Greenham, Marla Sullivan, Khalid Bazaid, Sinthuja Suntharalingam, Lana Silbernagel, Katherine Magner, Rébecca Robillard

**Affiliations:** 1 Mental Health Program, Children’s Hospital of Eastern Ontario, 401 Smyth Road, Ottawa, ON KH 8L1 Canada; 2 Department of Psychiatry, University of Ottawa, Ottawa, ON Canada; 3 Sleep Research Unit, The Royal’s Institute of Mental Health Research, Ottawa, ON Canada; 4 School of Psychology, University of Ottawa, Ottawa, ON Canada

**Keywords:** Sleep medications, Depression, Children, Adolescents

## Abstract

**Background:**

Primary care physicians and child and adolescent psychiatrists often treat sleep disturbances in children and adolescents with mood disorders using medications off-label, in the absence of clear evidence for efficacy, tolerability and short or long-term safety. This study is the first to report Canadian data about prescribing preferences and perceived effectiveness reported by child and adolescent psychiatrists regarding medications used to manage sleep disturbances in children and adolescents with depression.

**Methods:**

Canadian child and adolescent psychiatrists were surveyed on their perception of effectiveness of a range of medications commonly prescribed for sleep disturbances, their ranked preferences for these medications, reasons for avoiding certain medications, and perceived side effects.

**Results:**

Sixty-seven active child and adolescent psychiatrists completed the survey. Respondents reported noting significant sleep issues in 40% of all their patients. Melatonin and trazodone were identified as the first treatment of choice by 83% and 10% of respondents respectively, and trazodone was identified as the second treatment of choice by 56% of respondents for treating sleep disturbances in children and adolescents with depression. Melatonin (97%), trazodone (81%), and quetiapine (73%) were rated by a majority of respondents as effective. Doxepin, zaleplon, tricyclic antidepressants, zolpidem, or lorazepam were rarely prescribed due to lack of evidence and/or concerns about adverse effects, long-term safety, suitability for youth, suicidality, and dependence/tolerance.

**Conclusions:**

Melatonin and certain off-label psychotropic drugs are perceived as being more effective and appropriate to address sleep disturbances in children and adolescents with depression. More empirical evidence on the efficacy, tolerability and indications for using these medications and newer group of sleep medications in this population is needed.

## Background

Changes in sleep and biological rhythms emerge during childhood and adolescence [1, 2]. This developmental period also marks the onset of mood disorders for many individuals. Pediatric depression is characterized by prolonged or recurrent sadness or irritability, markedly diminished interest or pleasure in activities, decrease or increase in appetite, feelings of restlessness or being slowed down, poor memory and concentration, feelings of worthlessness and guilt, recurrent thoughts of death, as well as sleep disturbances [3]. Its prevalence increases in the early teens, more so in girls than boys [4]. A survey of Canadians aged 15 to 24 years indicated that about 11% have experienced depression in their lifetime, and 7% reported depression in the previous year [5]. In childhood and adolescence-onset depression, the risk of recurrence is high, about 50 to 70% within 5 years, and persisting depression is associated with worse suicidality [6]. Suicide is one of the leading cause of death among Canadian adolescents [7], and rates of suicide are increasing in this age group [8]. Poor sleep has been linked to worse and more recurrent depression, as well as increased suicidal ideation in adolescents [9]. Yet, little is known about how sleep disturbances co-occurring with depression are addressed in current pedopsychiatric practice.

Sleep disturbances are estimated to affect 66% to 72% of children and adolescents with depression [10, 11], and sleep loss was found to predict higher risks of depression in young Canadians [12]. These sleep problems can interfere with antidepressant response to standard treatments [13]. For instance, it is suspected that medications prescribed for depression may not be better than placebo in patients who also have sleep disturbances [14]. Furthermore, residual sleep problems after depression remission increase the risk of relapse [15, 16], and treating sleep disturbances in youths can improve depression [17, 18].

The use of medications prescribed for adult depression in youth is controversial; with concerns of modest therapeutic effects and higher risks for side effects, leading to questionable overall benefits-to-risk ratios [19, 20]. There is even less clarity for medications targeting sleep and circadian rhythms. Although there is no official approval, indication, or dosing guidelines for their use in children and adolescents, off-label treatment of sleep disturbances with over-the-counter and prescription medications are common [21]. There is inadequate information about the short-term and long-term tolerability, safety, and efficacy of sleep promoting medications and products in youth [22, 23]. As such, the Federal Drug Agency in the United States of America (FDA) does not approve the use of sleep promoting medications and products under the age of 18 years, and Health Canada only approved the use of diphenhydramine.

Considering the paucity of empirical evidence in pediatric populations, observational data of current practice and clinical impressions represent an important source of information. A study investigating prescription habits of Canadian general practitioners and pediatricians for children and adolescents with sleep problems reported that melatonin, antihistamines, antidepressants and even benzodiazepines were the most commonly used [24]. However, prescription habits to address sleep difficulties may differ in the context of pediatric mood disorders. This is especially relevant since children and adolescents seeing a psychiatrist are 3.6 times more likely to be prescribed a sleep medication than those seeing a general/family practice physician [25]. A national survey in the United States reported that trazodone was the most commonly prescribed sleep medication by psychiatrists for children with mood or anxiety disorders [23]. However, this study is now over a decade old and an update on current clinical knowledge and experience is warranted.

The current study aims to make use of clinical experience to generate more information about pharmacological sleep treatments in the context of pediatric depression. It reports on prescribing preferences and perceived effectiveness reported by Canadian child and adolescent psychiatrists regarding their use of medications for managing sleep disturbances in children and adolescents with depression.

## Methods

### Design and study population

A 16-item survey was sent to 433 members of the *Canadian Academy of Child and Adolescent Psychiatry* (CACAP) between October and December 2016. A follow-up letter urging participation was sent 4 weeks after the initial mail-out. The opportunity to enter a draw for a 1-year CACAP membership (valued at $325) or the registration fee to the annual CACAP conference (valued at $450) were offered as incentives. The Children’s Hospital of Eastern Ontario Research Ethics Board approved the study.

### Survey instrument

The survey designed by the research team was available in English and French, and could be filled either on paper or on REDCap [26]. Prior to distribution, it was piloted with a small number of child psychiatrists for clarity of content and readability. A copy of the final survey items is provided in Additional file 1.

Demographic questions (gender, years of active clinical practice, location of services, characteristics of the work setting, faculty appointment, and frequency of sleep disturbance in child and adolescent patients) were based on previous surveys from the College of Family Physicians of Canada, the Canadian Medical Association, and the Royal College of Physicians and Surgeons of Canada. The remaining questions were specifically designed to investigate prescribing preference and perceived effectiveness to treat sleep disturbances in children and adolescent patients with depression with the following sleep promoting medications: antihistamines, doxepin, lorazepam, other benzodiazepines, melatonin, mirtazapine, quetiapine, trazodone, tricyclic antidepressants, tryptophan, zaleplon, zolpidem, zopiclone, and herbals (e.g., valerian, lavender). Specifically, these questions asked about: (a) perceptions of effectiveness for each medication; (b) first and second prescription choices; (c) medication(s) never prescribed (indicating the reason by selecting one of the following options: lack of effect, concerns in youth, off label status, adverse effects, agitation, suicidality, long term safety, dependence or tolerance, lack of evidence); and (d) most common side effect observed (excessive sedation, daytime fatigue, nightmares or dreaming, agitation, dizziness, headache, memory impairment, postural orthostatic or tachycardia effects, or not applicable/do not use).

### Exclusion criteria

Surveys were systematically excluded for respondents who: were not child or adolescent psychiatrists, had not seen a child or adolescent patient within the previous 12 months, or filled out less than 60% of the items.

### Statistical analyses

For descriptive purposes, medians and standard deviations (or interquartile range (IQR) for skewed values) were calculated for continuous variables, and categorical variables were summarized using percentages and frequencies. Chi-square analyses were conducted to compare differences in the proportions of psychiatrists rating the main types of medications as first and second choices stratified by years of practice and practice settings. Data was analyzed using IBM SPSS Statistics for Windows [27].

## Results

### Sample characteristics

Of the 433 surveys mailed, 74 were returned (17.1% response rate). Of this sample, 67 (15.5%) surveys were cleared from the exclusion criteria. The majority of surveys were completed in English (94.0%). Sample characteristics are presented in Table 1.

**Table 1:** Sample characteristics

Gender (% female)	53.7
Years of active clinical practice (%)	
Less than 2 years	4.5
2 to 5 years	9.0
6 to 10 years	14.9
11 to 20 years	32.8
More than 20 years	38.8
New patients per week (Mdn)	4 (IQR 2–8)
Patients with significant sleep difficulties (Mdn %)	40.0 (IQR 23.8–52.5)
Province/territory (%)	
Nova Scotia	6.0
New Brunswick	1.5
Quebec	10.4
Ontario	49.3
Manitoba	4.5
Saskatchewan	3.0
Alberta	7.5
British Columbia	17.9
Others	0
Primary work setting (%)	
Academic/Tertiary Care Hospital	46.3
Community Hospital	13.4
Community Health Centre	11.9
Private practice	16.4
Other/multiple settings	10.4
Primary service type (%)	
Inpatient services	10.4
Outpatient services	61.2
Mix of both inpatient and outpatient	26.9
Type of community (%)	
Urban/suburban	88.1
Rural/small town	10.4
Remote	0
Faculty appointment (% yes)	77.6

*Mdn* median, *province/territories*—*others* Northwest Territories, Newfoundland/Labrador, Prince Edward Island, Nunavut, Yukon

### Perceived effectiveness and ranked preferences to treat sleep disturbances

Melatonin was perceived by 97.0% of respondents as effective, followed closely by trazodone (81.5%) and quetiapine (73.8%) (Table 2). Mirtazapine (55.4%) and zopiclone (52.3%) were also perceived to be effective by about half of respondents. Respondents most frequently reported melatonin and trazodone as their first and second choices respectively. Most respondents indicated that they never prescribe doxepin (87.9%), zaleplon (86.6%), tricyclic antidepressants (83.3%), zolpidem (77.6%), or lorazepam (68.2%) to treat sleep disturbances in pediatric depression.

**Table 2:** Perceived effectiveness and prescribing preferences

Medication	Find it effective (%)	First choice for treatment (%)	Second choice for treatment (%)	Never prescribe (%)
Melatonin	97.0	83.3	3.1	3.0
Trazodone	81.5	10.6	56.9	12.1
Quetiapine	73.8	0.0	12.3	24.2
Mirtazapine	55.4	1.5	6.2	31.8
Zopiclone	52.3	1.5	4.6	52.3
Other benzodiazepines	25.4	0.0	1.5	54.5
Antihistamines	23.4	0.0	0.0	60.6
Lorazepam	19.4	0.0	1.5	68.2
Tricyclic antidepressants	16.9	0.0	0.0	83.3
Tryptophan	16.9	3.0	4.6	65.7
Doxepin	9.5	0.0	1.5	87.9
Zolpidem	7.8	0.0	1.5	77.6
Herbals	6.3	0.0	0.0	62.1
Zaleplon	1.6	0.0	0.0	52.3

%: Percentage of respondents endorsing each medication type as being effective, being their first or second choice of treatment, or as a drug that they never prescribe for sleep difficulties in children and adolescents with depression

**Fig. 1:**
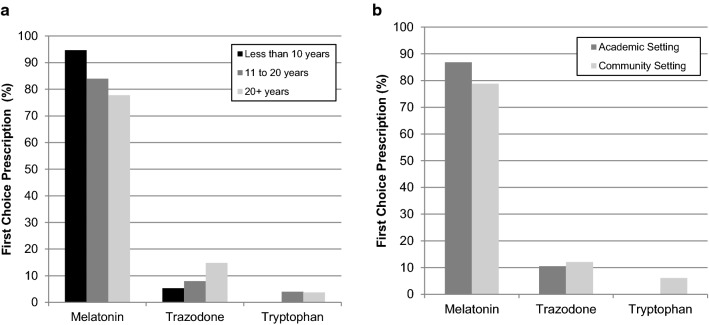
First choice prescriptions. Percentages of respondents who rated melatonin, trazodone and tryptophan as first choice for treatment of sleep disturbance in children and adolescents with depression as a function of years of clinical experience and practice setting

**Fig. 2:**
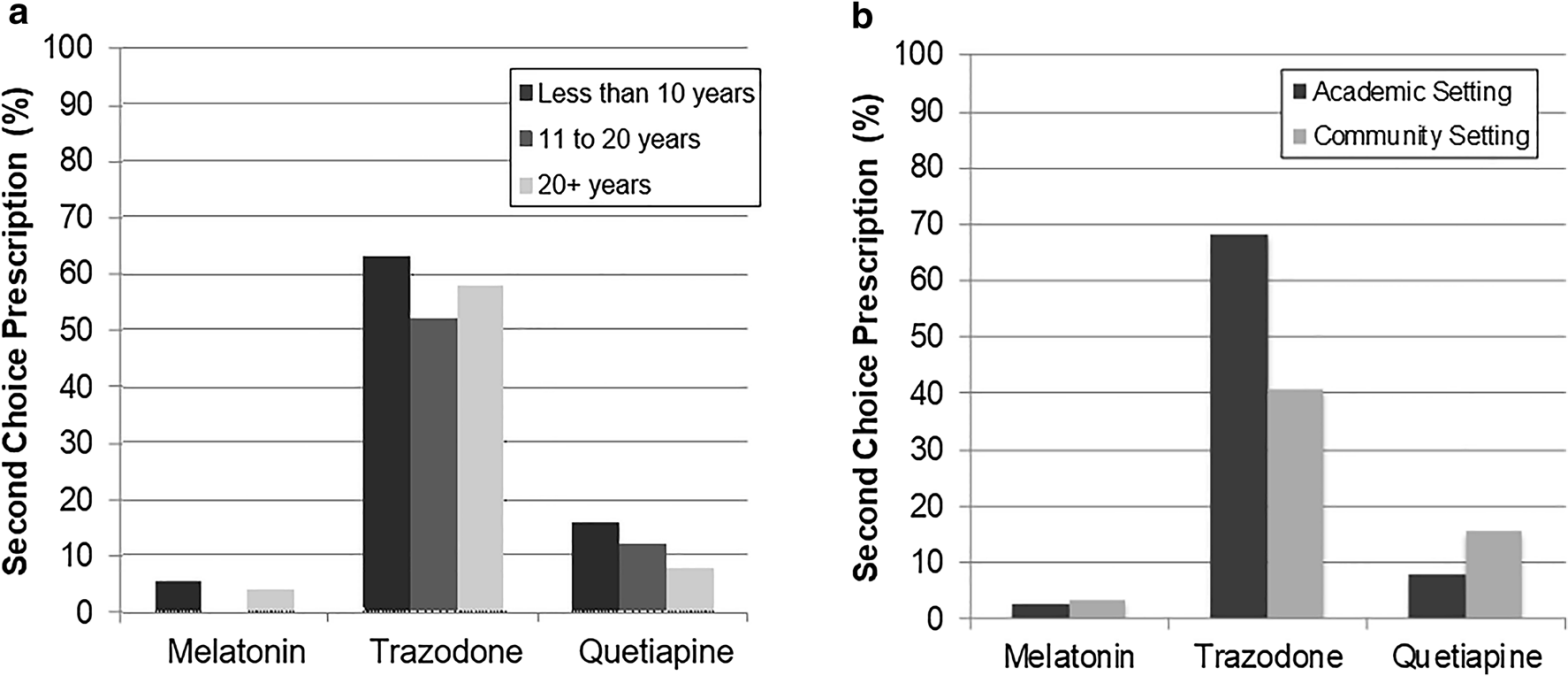
Second choice prescriptions. Percentages of respondents who rated melatonin, trazodone and tryptophan as second choice for treatment of sleep disturbance in children and adolescents with depression as a function of years of clinical experience and practice setting

The proportion of respondents who identified melatonin as their first choice decreased with the number of years of experience, and the opposite pattern was found for trazodone (Fig. 1a). There was no significant difference in the proportion of respondents identifying melatonin as second choice or trazodone as a first or second choice across subgroups based on years of experience (Fig. 2a, all p > 0.050).

There was no significant difference in rates of first choice medications between respondents from academic/tertiary settings compared to those from community settings (all p > 0.050, Fig. 1b). There was a significantly higher proportion of respondents who identified trazodone as their second choice in academic/tertiary settings compared to community settings (χ^2^(1) = 5.4, p = 0.020; Fig. 2b). No other difference in rates of second choice medications based on respondents’ settings reached statistical significance (all p > 0.050).

### Perceived risks and side effects

Table 3 reports perceived side effects for the most frequently preferred medications. For melatonin, the most commonly reported perceived side effects by respondents were: nightmares (16.4%), headache (9.0%), and fatigue (9.0%). Trazodone was frequently perceived to cause excessive sedation (37.3%) and fatigue (35.8%). These results were similar for quetiapine, with greater perceived negative side effects of excessive sedation (58.2%) and daytime fatigue (49.3%). Of note, 22.4% of respondents reported having concerns regarding the long-term safety of quetiapine.

**Table 3:** Perceptions about side effects

	Nightmares (%)	Daytime fatigue (%)	Headache (%)	Excessive sedation (%)	Long-term safety (%)
Melatonin	16.4	9.0	9.0	3.0	4.5
Trazodone	11.9	35.8	13.4	37.3	6.0
Quetiapine	4.5	49.3	3.0	58.2	22.4

%: Percentages of respondents who noticed various side effects when prescribing melatonin, trazodone and quetiapine (i.e. the three medications which were rated by the most respondents as effective)

As for medications reported as the least frequently prescribed (doxepin, zaleplon, tricyclic antidepressants, zolpidem, and lorazepam), most respondents reported they avoided them because of: dependence or tolerance, concerns about their use in youth, adverse effects, lack of evidence, and long-terms safety concerns (see Table 4).

**Table 4:** Reported concerns

	Long-term safety (%)	Adverse effects (%)	Lack of evidence (%)	Concerns in youth (%)	Dependence/tolerance (%)	Suicidality (%)
Doxepin	22.4	31.3	22.4	17.9	3.0	3.0
Zaleplon	16.4	20.9	22.4	35.8	29.9	0.0
Tricyclics	22.4	59.7	22.4	37.3	1.5	20.9
Zolpidem	16.4	20.9	20.9	34.3	32.8	0.0
Lorazepam	29.9	31.3	10.4	38.8	76.1	6.0

%: Percentages of respondents who reported various concerns about the less commonly prescribed medications

## Discussion

This is the first report about prescribing preferences and perceived effectiveness reported by Canadian child and adolescent psychiatrists regarding the use of sleep medications in children and adolescents with depression. The results indicate that a majority of Canadian child and adolescent psychiatrists perceive melatonin, trazodone, and quetiapine as effective in treating sleep disturbances in that population. Specifically, melatonin and trazodone were identified as the first treatment of choice by 83% and 10% of respondents respectively, and trazodone was identified as the second treatment of choice by 56% of respondents. Melatonin was also perceived as having the least side effects. While there is a pressing need for empirical data on this topic, these findings derived from clinical experience increase the knowledge base on pharmacotherapies for the management of sleep problems in the context of pediatric depression.

Over 10 years ago, a study on American psychiatrists reported that trazodone was the most commonly prescribed sleep medication for children with mood and anxiety disorders, reaching a prescription rate of 78%, while alpha-2 agonists were prescribed 40% of the time [23]. Conversely, melatonin was recommended by a third of respondents. A more recent report on Ontario pediatricians and family physicians noted that melatonin (73%), over-the-counter antihistamines (41%), antidepressants (37%), and benzodiazepines (29%) were the most commonly recommended medications [24]. Overall differences in prescription preferences between the current findings and these past studies suggest that melatonin is increasingly used to address sleep problems in youths with depression.

The current results suggest favorable prescribers’ perceptions about melatonin in terms of efficacy and side effects, which may be slightly more prominent in younger generations of practitioners. This is aligned with emerging evidence, notably in Europe, that melatonin use in children and adolescents has been increasing over time [28]. Empirical data on the effects of melatonin in clinical pediatric population is scarce. An observational study in 100 children with disabilities receiving melatonin for chronic sleep disturbances suggested sleep improvements in 80% of that sample, without major side effects or signs of tolerance [29]. Furthermore, a melatonin agonist administered about 2 h before bedtime induced a phase advance in endogenous melatonin release, and improved both sleep and mood in adolescents and young adults with depression [18]. Although melatonin is considered safe [30], concerns persist about long-term safety in children and adolescents, notably for the timing of puberty and potential interferences with the menstrual cycle [31, 32]. Further work is required to determine how the potential side effects of melatonin may compare to those of classical sleeping medications in children and adolescents with depression. Also, the observation that melatonin is increasingly used to address sleep issues in youths stress the importance of tighter regulations since inconsistencies in concentration and active ingredients in melatonin tablets have been reported [33].

In the current study, aside from melatonin, trazodone and quetiapine were most commonly perceived as effective. Although it was initially developed for the treatment of depression, trazodone has become the most commonly prescribed medication for insomnia [34]. These trends in adult prescriptions, and concerns regarding the tolerance and dependence resulting from hypnotics and benzodiazepines use, may have influenced child and adolescent psychiatrists in their prescription habits. In Canada, quetiapine has been approved for schizophrenia, bipolar disorder and treatment-resistant major depressive disorder. Low doses of quetiapine are also commonly prescribed for sleep disorders, although this indication has not been officially approved and concerns were raised about potential adverse effects (e.g. fatal hepatotoxicity, QT prolongation and akathisia). Nevertheless, the perceived efficacy of these two drugs based on clinical observations reported in the current study suggest that they may be relevant medications to investigate further in children and adolescents with depression and sleep complaints.

In the current study, the majority of respondents reported never prescribing antihistamines for insomnia in depressed youth, but 22% did report finding it to be effective. The latter conflicts with studies showing that H1 antihistamines are no more effective than placebo for adult insomnia [35]. Similarly, in infants, diphenhydramine is no more effective for sleep than placebo [36]. These medications are thought to induce tolerance [35], and pose risks for overdose in children, either alone or in combination with other over-the-counter allergy or cold preparations.

Practitioners in the present study generally avoided benzodiazepines and non-benzodiazepine hypnotics, both of which have worrisome adverse effects. While there is regulatory approval for these drugs in adults, they are not approved for use in children and adolescents, and respondents expressed several concerns about their use in depressed youth. In other words, Canadian child and adolescent psychiatrists were not more favorable to the use of medications which have regulatory approval for adults. Whether this is a justifiable practice remains an open question.

Newer sleep promoting medications not yet approved in Canada are worth mentioning. For example, agomelatine has approval for the treatment of major depressive disorder in Australia and the European Union, but not in Canada or the United States [37]. Ramelteon, tasimelteon, targeting the non-24-h-sleep–wake disorder, and suvorexant, an orexin receptor antagonist, are approved in the USA but not in Canada. At low-dose, doxepin, a tricyclic antidepressant with potent antihistaminergic effects, is approved in Canada and the United States, but not for youths. Anticonvulsant drugs, such as gabapentin and pregabalin, are not officially approved for sleep but may increase slow wave sleep and attenuate sleep disturbance [38–40]. While clinical trials with these newer agents in children and adolescents are likely to remain limited, it will be important to collate clinical observations on their evolving off-label use in clinical settings.

Several study limitations should be noted. We do not know if the rather large proportion of our respondents holding a faculty position is representative of most Canadian child and adolescent psychiatrists, a factor likely to limit the generalizability of our findings. The low response rate may limit generalizability, however it is comparable to the response rate of other Canadian and American survey studies looking at prescribing habits of sleep medications for pediatric patients [18, 24]. There was no open question or qualitative methods used as part of this survey. Importantly, this study did not consider whether children and adolescents with sleep disturbance and depression share the views of their psychiatrists with respect to the effectiveness or safety of these sleep promoting medications. Children, adolescents and their families often prefer nonpharmacological approaches over pharmacotherapies for the management of sleep and depressive symptoms [41], but nonpharmacological treatments of sleep disturbance were not addressed in the survey. Such interventions, like cognitive behavioural therapy for insomnia, have been found to be more beneficial than frequently used medications in children, adolescents and adults [42]. Future surveys documenting common clinical practices to address sleep problems in pediatric depression should investigate potential barriers to nonpharmacological interventions (availability, cost, and paucity of trained practitioners).

## Conclusion

Melatonin and trazodone were the medications indicated by the highest number of Canadian child and adolescent psychiatrists surveyed as being effective and as being their first or second choice of treatment for pediatric sleep disturbances in the context of depression. Sleep promoting medications, including the newer generation of drugs, may have a role in the treatment of sleep disturbance in depressed youth, but empirical data from clinical trials to assess efficacy, tolerability and long-term effects are needed. The American Academy of Sleep Medicine guidelines for the management of insomnia propose nonpharmacological approaches as the first line treatment in adults, and there is increasing evidence supporting applications of behavioural and psychotherapeutic approaches in youth. There is a need to assess how much this is actually integrated in common clinical practice. Such interventions are yielding promising effects for both sleep and mood in youth with depression.

## Supplementary information

**Additional file 1.** CACAP survey questions.

## Data Availability

All the data and materials of the project are available upon request.
