# Supplementary material for: Medications for sleep disturbance in children and adolescents with depression: a survey of Canadian child and adolescent psychiatrists
Source: Child Adolesc Psychiatry Ment Health. 2020 Mar 10;14:10. doi: 10.1186/s13034-020-00316-8 (PMC7063733; doi:10.1186/s13034-020-00316-8)
Supplement: Supplementary file 1 — Additional file 1. CACAP survey questions. [file 13034_2020_316_MOESM1_ESM.docx]

Additional file 1: CACAP survey questions

1. What is your gender?
2. How many years have you been in active clinical practice as a child and adolescent psychiatrist?
3. Have you provided clinical care to children and adolescents in the past year?
4. On average, how many new patients do you see each week?
5. In which province/territory do you provide clinical care?
6. What is your primary work setting?

(Academic or Tertiary Care Hospital/Community Hospital/Community Health Centre/ Private Practice/Other)

1. Which type of service best characterizes your primary patient care?

(Inpatient services/Outpatient services/Mix of both inpatient and outpatient)

1. In which type of location do you provide clinical care in your primary work setting?

(Urban or Suburban/Rural or Small town/Remote)

1. Do you hold a university faculty appointment?
2. Approximately what percentage (%) of children and adolescents in your practice has significant sleep difficulties?
3. Thinking about your actual practice over the past year, do you find the following medications^1^ to be effective to treat sleep disturbance in depressed children and adolescents? PLEASE INDICATE FOR EACH MEDICATION.
4. We acknowledge that sleep hygiene approaches are very often recommended before medication is considered. Thinking about your actual practice over the past year, and how you are currently treating sleep disturbance in depressed children and adolescents, please indicate which of the following^1^ you would be most likely to prescribe as INITIAL medication therapy. PLEASE SELECT ONE.
5. Thinking about your actual practice over the past year, if your first choice of medication for sleep was ineffective or poorly tolerated, which of the following medications^1^ would you be most likely to prescribe NEXT for sleep disturbance in depressed children and adolescents? PLEASE SELECT ONE.
6. Which of these medications^1^ do you never prescribe for depressed children and adolescents for sleep difficulties? SELECT ALL THAT APPLY.
7. If you avoid prescribing certain medications to treat sleep disturbance in depressed children and adolescents, please indicate your reason why^1^: PLEASE CHECK EACH BOX THAT APPLIES.

(Lack of effect/Concerns in youth/Off label status/Adverse effects/Agitation/Suicidality/Long term safety/Dependence or tolerance/Lack of evidence)

1. Thinking about your actual practice over the past year, what side effects do you see more commonly associated when the following medications^1^ are used in depressed children and adolescents.

(Excessive sedation/Day-time fatigue/Night-mares or dreaming/Agitation/Dizziness/Head-ache/Memory impairment/Postural orthostatic or tachycardia effects/Not applicable/Don’t use)

Note. ^1^Questions 11-16 refer to the following medications: Antihistamines, Doxepin, Herbals (e.g., Valerian, Lavender), Lorazepam, Other Benzodiazepines (e.g., oxazepam, temazepam, flurazepam, triazolam, alprazolam, clonazepam), Melatonin, Mirtazapine, Quetiapine, Trazodone, Tricyclic Antidepressants (i.e., amitriptyline, nortriptyline), Tryptophan (i.e., 5-HTP), Zaleplon, Zolpidem, Zopiclone, Other
